# Synthesis and characterization of cost-effective and high-efficiency biochar for the adsorption of Pb^2+^ from wastewater

**DOI:** 10.1038/s41598-023-42918-0

**Published:** 2023-09-20

**Authors:** Hatef Bassareh, Masoud Karamzadeh, Salman Movahedirad

**Affiliations:** Department of Chemical Engineering, Iran University of Science and Technology, Tehran, Iran

**Keywords:** Environmental impact, Biosynthesis, Chemical engineering

## Abstract

This study aimed to investigate the adsorption mechanism of Pb^2+^ in wastewater using activated carbon derived from inexpensive materials, specifically avocado, bitter orange, and walnut leaves, through a single-step chemical activation process. The activated carbon was prepared using sulfuric acid as an activator, with a particle size of 1 mm. The pyrolysis reactor (slow-pyrolysis) operated at 600 °C for 90 min with a nitrogen flow rate of 5 L/min. Batch experiments were conducted under various conditions to determine the optimal dosage (1.5 g/L), equilibrium contact time (180 min), and pH (6.5). The study focused on employing cost-effective and highly efficient adsorbents, namely biochar produced from tree leaves, for the adsorption process. The results indicated that the pseudo-second-order kinetic model accurately described the adsorption process, while the Freundlich isotherm model best fit the experimental data. These findings suggest that tree leaves can serve as cost-effective and efficient adsorbents for a wide range of applications. Furthermore, multiple adsorption factors were evaluated in batch mode, including contact duration, pH, adsorbent dosage, concentration of the Pb^2+^ solution, and temperature. The maximum adsorption capacities for the activated carbon derived from avocado, bitter orange, and walnut leaves were found to be 60.46, 59.42, and 58.48 mg/g, respectively. Thus, this study highlights the effectiveness and economic feasibility of using pyrolysis-derived activated carbon from low-cost materials for the removal of Pb^2+^ from wastewater.

## Introduction

In recent decades, the water quality has been affected by increasing urbanization, increasing population and development of industries. Heavy metals are one of the most hazardous pollutants which cause serious environmental and health problems owing to their toxicity, biodegradability, and accumulation^1, 2^. Pb^2+^, in particular, is one of the most hazardous heavy metals found in the effluents of various industries such as paint, battery, and printing and can cause severe damage to the kidneys, liver, and brain^3, 4^. Therefore, the Pb^2+^ removal process from wastewater before discharging it into the environment is essential for such industries (The acceptable level of Pb^2+^ in the industrial wastewater by DoE (Department of Environment.) is 0.1 ppm and by WHO is 0.01 ppm)^5, 6^.

Various techniques are available for removing heavy metal ions, including precipitation, flocculation, reduction, ion exchange, evaporation recovery, elecroFenton, persulfate process, Fenton process, Nano photocatalytic system, liquid–liquid extraction, electrocoagulation, adsorption, and membrane processes^7–20^. Adsorption offers several advantages including cost-effectiveness, high efficiency in removing pollutants, and ease of operation compared to the mentioned techniques^21–23^. Various models have been used for examining the adsorption equilibrium data. Isothermal model parameters and their thermodynamic assumptions offer a deeper understanding of the adsorption mechanism, surface properties, and adsorbent affinity. Although most of these models are dependable for small relative pressures, they are not as accurate when tested in a broader range. Adsorption isotherms can be classified into two categories: two-parameters and three-parameters isotherms. Meanwhile, adsorption kinetics measures the adsorption capacity over time^24–28^.

The adsorbent selection is crucial in the adsorption process, and activated carbon is a commonly utilized adsorbent. Activated carbon has been used in the adsorption process for over a century. The usage of activated carbon as adsorbent can be traced back to the early 1900s when it was employed as a decolorizing agent in the sugar industry. Since then, its applications have expanded to various fields, including water treatment, air purification, and gas separation. According to the international biochar initiative (IBI), activated carbon is a solid substance derived from the thermal conversion and decomposition of biomass into charcoal. Activated carbon can be categorized in various ways based on different factors, including organic and inorganic biomass, primary or secondary biomass, and others^29–34^.

Numerous studies have been conducted to explore the application of the biomass because of the cost-effectiveness and environmentally friendly nature of the activated carbon. Alkherraz et al.^35^, investigated the adsorption of Pb^2+^, zinc, copper, and cadmium using an adsorbent derived from the pyrolysis of olive tree branches with phosphoric acid as the chemical activator. According to their findings, the adsorbent demonstrated adsorption capacities of 41.32, 34.97, 43.10, and 38.17 mg/g for Pb^2+^, zinc, copper, and cadmium, respectively. Babel et al.^36^, conducted a study to explore adsorbents with high capacity for removing heavy metals. The investigated adsorbents included chitosan (with adsorption capacities of 815, 273, and 250 mg/g for Pb^2+^, Cr^6+^, and Cd^2+^, respectively), zeolites (with adsorption capacities of 175, 137 mg/g for Pb^2+^, and Cd^2+^, respectively), and waste slurry (with adsorption capacities of 1030, 560, and 540 mg/g for Pb^2+^, Cr^6+^, and Cd^2+^, respectively). Ashrafi et al. investigated the capacity of the synthesized core–shell nanoparticles for the adsorption of Pb^2+^. They studied the effects of different factors on the adsorption process, including the contact time, pH of the solution, adsorbent quantity, initial Pb^2+^ concentration, and temperature^5^. Their results illustrated that, the adsorption occurred rapidly, and the equilibrium was reached within approximately 2 h. Furthermore, the results demonstrated that the Langmuir isotherm exhibited the best fit for the equilibrium data, indicating a maximum adsorption capacity of 666.67 mg/g for the adsorbent. Kadirvelu et al.^37^, conducted a study to determine the applicability of coir pith carbon in eliminating heavy metals from industrial effluents by varying the pH and carbon concentration parameters. The adsorption capacities of the adsorbent were found to be the highest at 450, 300, 300, 250, and 125 mg/g for copper ions (Cu^2+^), Pb^2+^ ions (Pb^2+^), cadmium ions (Cd^2+^), nickel ions (Ni^2+^), and mercury ions (Hg^2+^), respectively. Karnib et al.^38^ observed that activated carbon could adsorb more than 90% of nickel in their study. According to Seyfi Hasankola et al.^39^, an iron-based metal–organic framework adsorbent containing a ligand called (${H}^{2+}-TCPP$) was able to adsorb mercury (Hg^2+^) with a capacity of 233 mg/g. Wang et al.^40^ studied the combination of chitosan and an organic–metallic framework as a novel adsorbent for the removal of heavy metal ions. Their results demonstrated that the maximum adsorption capacity of chromium (Cr^6+^) was 93.6 mg/g at pH 2 and 40 °C after 8 h of adsorption. In addition, under the conditions of pH 5 and 60 °C, the adsorbent exhibited a copper (Cu^2+^) adsorption capacity of 50.6 mg/g, whereas the nickel (Ni^2+^) adsorption capacity was found to be around 60 mg/g at a pH of 5 and a temperature of 20 °C.

In this study, we aimed to produce biomass and investigate its potential as an effective and sustainable method for removing heavy metals from contaminated water. Biomass, including lignin, cellulose, and hemicellulose, undergoes thermal decomposition, causing temperature elevation as the components decompose. However, identifying the specific component of biomass that contributes to improve the adsorption properties is challenging, as it depends on various factors such as surface hardness, porosity, and functional groups. So, this study involves the comparison of the adsorption capacity of three adsorbents derived from the low-pyrolysis of avocado, walnut, and Bitter orange leaves, in removing the Pb^2+^ (as a heavy metal) from the contaminated water. To ensure accurate and reliable comparisons, optimal values for parameters such as synthesis temperature, retention period, nitrogen flow rate, the rate of temperature change, heavy metal concentration, and particle size were determined through various experiments. In sum, the objective of this study was to produce, evaluate and compare the adsorption capacities of three biochar adsorbents for Pb^2+^ removal using a batch adsorption process.

## Experimental process

### Chemicals and experimental setup

This study utilized Bitter orange, avocado, and walnut tree leaves from Mazandaran, Iran to produce activated carbon. Table 1, illustrated the lignocellulosic composition and structural variances of these materials. The choice of these three biomasses was grounded on variations in their lignocellulosic structures and their capacity to adsorb the Pb^2+^, which were compared. The Pb (II) nitrate salt (Pb(NO_3_)_2_) was purchased from Merck (Germany).

**Table 1:** Lignocellulosic composition of three biomasses of avocado, Bitter orange, and walnut^41^.

Biomass	Lignin (%)	Cellulose (%)	Hemicellulose (%)	Ash (%)
Avocado leaf	1.79	6.48	47.88	7.02
Bitter orange leaf	2.4	13.6	6.1	1.6
Walnut leaf	49.1	25.4	21.2	3.6

### Pyrolysis setup

A fixed-bed pyrolysis reactor was designed to produce biocarbon from biomass. The reactor is a vertical, cylindrical, and fixed-bed type made of stainless steel with an inner diameter of 3.8 cm and height of 32 cm. The entry point for biomass and nitrogen gas is located at the top of the reactor. An electric furnace with a height of 30 cm and a power of 2 KW was used to provide the necessary heat for the pyrolysis. The exhaust gas from the reactor was directed to a copper condenser with a capacity of 9.95 kW. Some of the exhaust gas was liquefied and collected in a separate container, while the non-condensable gases were removed from the gas outlet section. This reactor design follows standard practices in the fixed-bed pyrolysis and utilizes nitrogen gas to minimize oxygen exposure and reduce the risk of combustion during the process^42^. The copper condenser is also a common feature, allowing for the recovery of valuable by-products such as bio-oil and water^43, 44^.

The slow pyrolysis system was utilized for its high efficiency in producing activated carbon. The reactor tests showed that the system can yield up to 50% of activated carbon, which is the highest yield. On the other hand, the lowest yield was for bio-oil, which is a liquid product. The furnace was powered by an electronic board, and the heat was transferred to the reactor. Nitrogen gas was introduced into the reactor from the top and exited from the bottom, passing through the biomass and the grid to reduce the biomass mass and cool down as it entered the gas condenser. Some of the gas were condensed at the end of the condenser, whereas the non-condensable gases were eliminated.

### Preparation of activated carbon

Activated carbon was produced from avocado, Bitter orange, and walnut leaves using a chemical activation process that includes the following steps:

In the first step, certain amounts of avocado, Bitter orange, and walnut leaves were dried in an oven at 110 °C for 12 h to be completely dry. The dried biomass was ground and sieved to collect a set of biomass samples of all three materials with a value of 2 mm. In the next step, the dried biomass was fertilized with a sulfuric acid solution in a ratio of 4:1 for 6 h in a vibrating oven. The mixture was then dried completely by keeping it at 120 °C for 48 h. Then, the fertilized dry matter underwent carbonization and chemical activation in a pyrolysis reactor, with a temperature rate of 20 °C/min until reaching 600 °C. The temperature was then held constant for 1.5 h under nitrogen gas with a flow rate of 0.5 L/min. The obtained activated carbon was washed several times with 0.5 M hydrochloric acid and then rinsed repeatedly with cold distilled water to bring the solution pH through the solid phase at about 6–7. Finally, the remaining solid was dried in an oven at 110 °C for 24 h to be ready for the next process.

### Batch adsorption

The synthesized wastewater with initial concentration of 50 mg/L was prepared by dissolving a suitable amount of Pb^2+^ in the distilled water (Considering the Molecular Weight of Pb^2+^ and Pb(NO3)2, which are 207.2 and 331.2 g/mol, respectively, in this research, 0.016 g of salt was used to prepare a 100 ppm solution, and then we diluted it to the desired amount). In order to investigate the initial concentration effect on the adsorption capacity, the concentrated wastewater was thinned with distilled water. Based on the literature regarding the elimination of heavy metals using activated carbon, a quantity of 0.15 g of adsorbent per 100 mL of synthesized wastewater was used in this study. Therefore, a dose of 1.5 g/L of adsorbent was employed in all experiments (With the exception of the experiments aimed at examining the influence of the adsorbent quantity). Additionally, all experiments were conducted in 250 mL Erlenmeyer flasks.

This study investigated various parameters that affect the removal of Pb^2+^, including contact time, solution pH, initial concentration of Pb^2+^ in wastewater, temperature, and adsorbent dosage. The classical method was employed, whereby one parameter was considered variable while the other parameters were held constant. Specifically, each experiment consisted of adding 0.15 g of adsorbent to 100 mL of a certain concentration of Pb^2+^ salt solution in a 250 mL Erlenmeyer flask at 25 °C (except for the temperature effect experiment) and stirring the mixture at 120 rpm (cooling incubator-CIT53, Teb Azma, Iran) until reaching equilibrium. The concentration of Pb^2+^ was determined using an Inductively Coupled Plasma Spectrometer (ICPS-7000, Shimadzu, Japan)^5^. It should be noted that the pH of the solutions was modified by adding small droplets (< 0.1 mL) of 0.5 M HCl and KOH solutions. One of the objectives of this study, is analyzing the Langmuir, Freundlich, and Temkin isotherms to investigate the equilibrium state and kinetics of an adsorption process in a system. In vitro data was utilized to apply the pseudo-first-order, pseudo-second-order, and intra-particle diffusion models to assess the kinetics of the adsorption process.

The quantities of adsorbed Pb^2+^ per unit mass of adsorbent (q_e_, mg/g) and the removal efficiency (%) were calculated by incorporating the initial concentration of the Pb^2+^ (C_0_), the equilibrium concentration of the Pb^2+^ (C_e_), the volume of the solution (V), and the weight of the adsorbent used in the study (M) as the following equations:

1 $$q_{{_{e} }} = (C_{0} - C_{e} )\frac{V}{M}$$

2 $$Removal\,efficiency \, \left( \% \right) = \frac{{C_{0} - C_{e} }}{{C_{0} }} \times 100$$

### Adsorption kinetics and equilibrium models

To provide a precise description of the adsorption kinetics for different adsorbate-adsorbent systems under varying experimental conditions, it is essential to compare the models' projected adsorption characteristics with experimental behavior. In this study, we applied three frequently employed kinetic models to analyze the data.

Lagergren expressed the pseudo-first-order velocity equation as Eq. (3)^45^:

3 $$\frac{{dq_{t} }}{dt} = k_{1} (q_{e} - q_{t} )$$

The rate constant of the equation (k_1_, 1/min) is computed by integrating Eq. (4) over the interval of t = 0 to t = t and q_t_ = 0 to q_t_ = q_t_, where q_e_ and q_t_ correspond to the adsorption capacity at equilibrium (mg/g) and the quantity of the adsorbed metal at time t (min), respectively.

4 $$\begin{aligned} & Log\left( {1 - \frac{{q_{t} }}{{q_{e} }}} \right) = - \left( {\frac{{k_{1} }}{2.303}} \right)t \\ & Ln(q_{e} - q_{t} ) = Lnq_{e} - k_{1} t \\ \end{aligned}$$

The values of q_e_ and k_1_ can be obtained by plotting ln (q_t_—q_e_) against t and calculating the intercept and slope of the resulting line, respectively^46^.

The adsorption of divalent metal ions on the rock was explained by Ho and McKay^47^. This model known as the pseudo-second order model and expressed as Eq. (5):

5 $$\frac{{dq_{t} }}{dt} = k_{2} (q_{e} - q_{t} )^{2}$$

The relationship between q_e_ and q_t_ can be described by Eq. (5). The equation's rate constant is represented by k_2_ (1/min), and it can be determined by integrating Eq. (3) over the interval of t = 0 to t = t and q_t_ = 0 to q_t_ = q_t_, resulting in a linear relationship as shown below:

6 $$\frac{t}{{q_{t} }} = \frac{1}{{k_{2} q_{e}^{2} }} + \frac{1}{{q_{e} }}$$

The values of q_e_ and k_2_ can be obtained by plotting *t*/*q*_*t*_ against t and calculating the intercept and slope of the resulting line, respectively^46^.

The intraparticle diffusion model's fundamental assumption is that the rate is exclusively determined by intra-particle diffusion, with film diffusion being excluded as a determining factor. The nonlinear equation for the intra-particle diffusion model is presented below:

7 $$q_{t} = k_{id} t^{0.5} + I$$

where *q*_*t*_ is the amount of adsorbate at *t* and k_i_ (mg/g.min^0.5^) is a constant intra-particle rate^48^.

In contrast, the thickness of the boundary layer is directly proportional to the value of *I*. When *I* equal zero, intra-particle diffusion is the sole limiting factor in the process. Furthermore, the system's slow and fast adsorption is denoted by negative and positive values of *I*, respectively^49^.

Adsorption isotherms are employed to characterize the equilibrium relationship between adsorbates and adsorbents. Various theoretical and empirical isotherms have been proposed in recent years. However, many of these models are only applicable to small pressure ranges and do not correspond well to the experimental data over a wider range^50, 51^. Nonlinear models, such as Langmuir's, Freundlich's, and Temkin's, have been utilized to investigate the adsorption equilibrium behavior.

8 $$q_{e} = \frac{{q_{m} K_{L} C_{e} }}{{1 + K_{L} C_{e} }}$$

9 $$q_{e} = K_{F} C_{e}^{1/n}$$

10 $$q_{e} = \left( {\frac{RT}{{b_{T} }}} \right)\ln (K_{T} C_{e} )$$

The Langmuir model employs q_m_ (mg/g) to represent the maximum adsorption capacity, while K_L_ (L/mg) is the Langmuir constant associated with the adsorption energy. The Freundlich isotherm employs K_F_ (mg^1-1/n^ L^1/n^/g) as the Freundlich constant, which indicates the adsorbent's relative adsorption capacity, with n (1) as the Freundlich equation exponent. The Temkin isotherm comprises b_T_ (kJ/mol) and K_T_ (L/mg), which respectively represent the Temkin constant related to the heat of sorption and the Temkin equilibrium isotherm constant^52–56^.

## Analysis and discussion of the results

### Characterization

To analyze the loading of functional groups on activated carbon, Fourier transform infrared (FTIR) spectroscopy was conducted using KBr tablets (Perkin Elmer Spectrum, RX1-Germany). The N_2_ gas adsorption method (BET) was employed at 300 °C to determine the activated carbon's porosity and specific surface area. Moreover, thermogravimetric analysis (TGA) was conducted to evaluate the mass reduction of the three biomasses with increasing temperature and assess the adsorbent's thermal stability.

**Figure 1:**
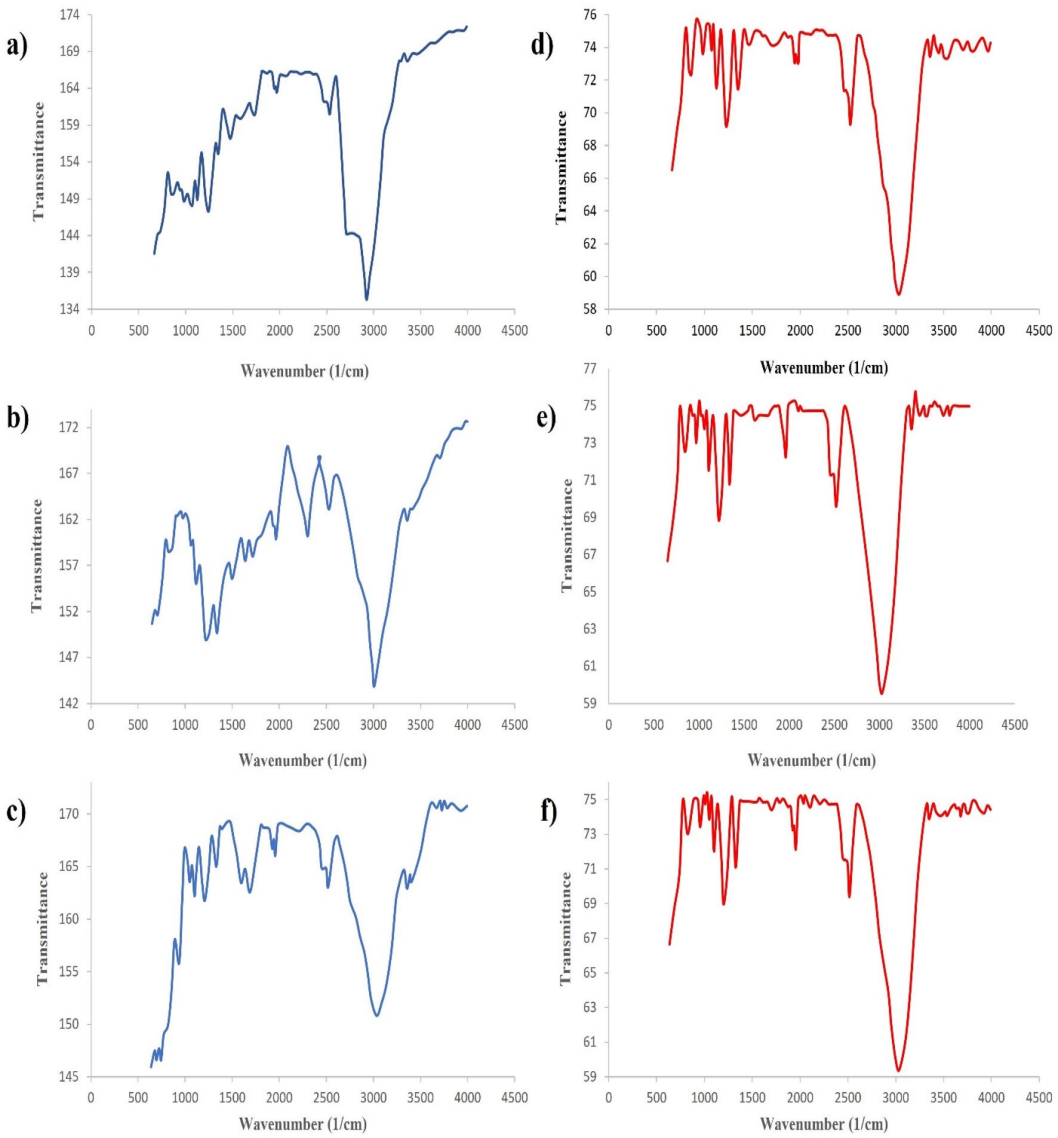
FTIR diagrams for (**a**) avocado, (**b**) bitter orange, (**c**) walnut before pyrolysis, (**d**) avocado, (**e**) bitter orange, and (**f**) walnut after pyrolysis.

Figure 1, depicts FTIR analyses conducted on avocado, Bitter orange, and walnut samples before and after pyrolysis, respectively. Table 2 presents the FTIR analysis results for Bitter orange, avocado, and walnut leaves before and after pyrolysis. The spectral measurements were performed within the peak range of 560 to 4400 cm^−1^.

**Table 2:** Functional group present in avocado, Bitter orange, and walnut leaves.

Functional group	Vibration	Functional	Peak
_–OH_	Stretch/scissoring	Lignin, cellulose and hemicellulose	3030/3300/3410
_–CH_	Stretch	cellulose and hemicellulose	2916/2800/874/2850
_C=O_	Stretch	Lignin and hemicellulose	1711/1611/1644/1585
_C=C_	Stretch	Lignin	1500/1611
_C–C_	Stretch	Lignin, cellulose and hemicellulose	1436
_C–O_	Stretch	Lignin, cellulose and hemicellulose	1045

After subjecting the raw biomass of avocado, Bitter orange, and walnut tree leaves to pyrolysis and its associated secondary reactions in the reactor, specific peaks that were initially observed in the raw samples have been eliminated. A noteworthy example is the avocado leaves, where peaks at wave numbers 3300, 1858, and 1436 representing –OH, C=O, and C–C bonds, respectively, have disappeared after the pyrolysis process^57–59^.

**Figure 2:**
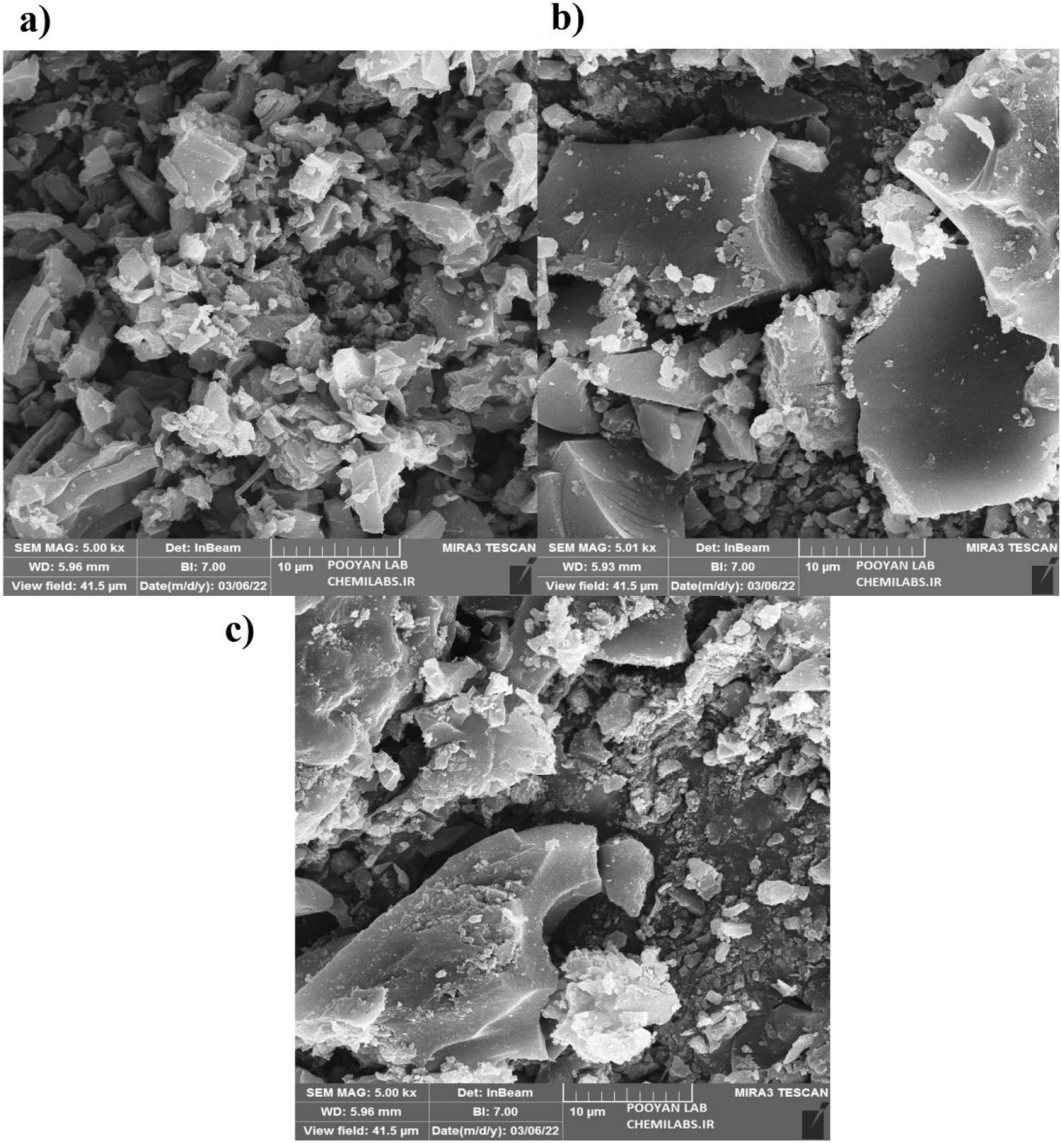
Image of (**a**) avocado, (**b**) Bitter orange and (**c**) walnut biochar from a distance of 10 µm.

**Figure 3:**
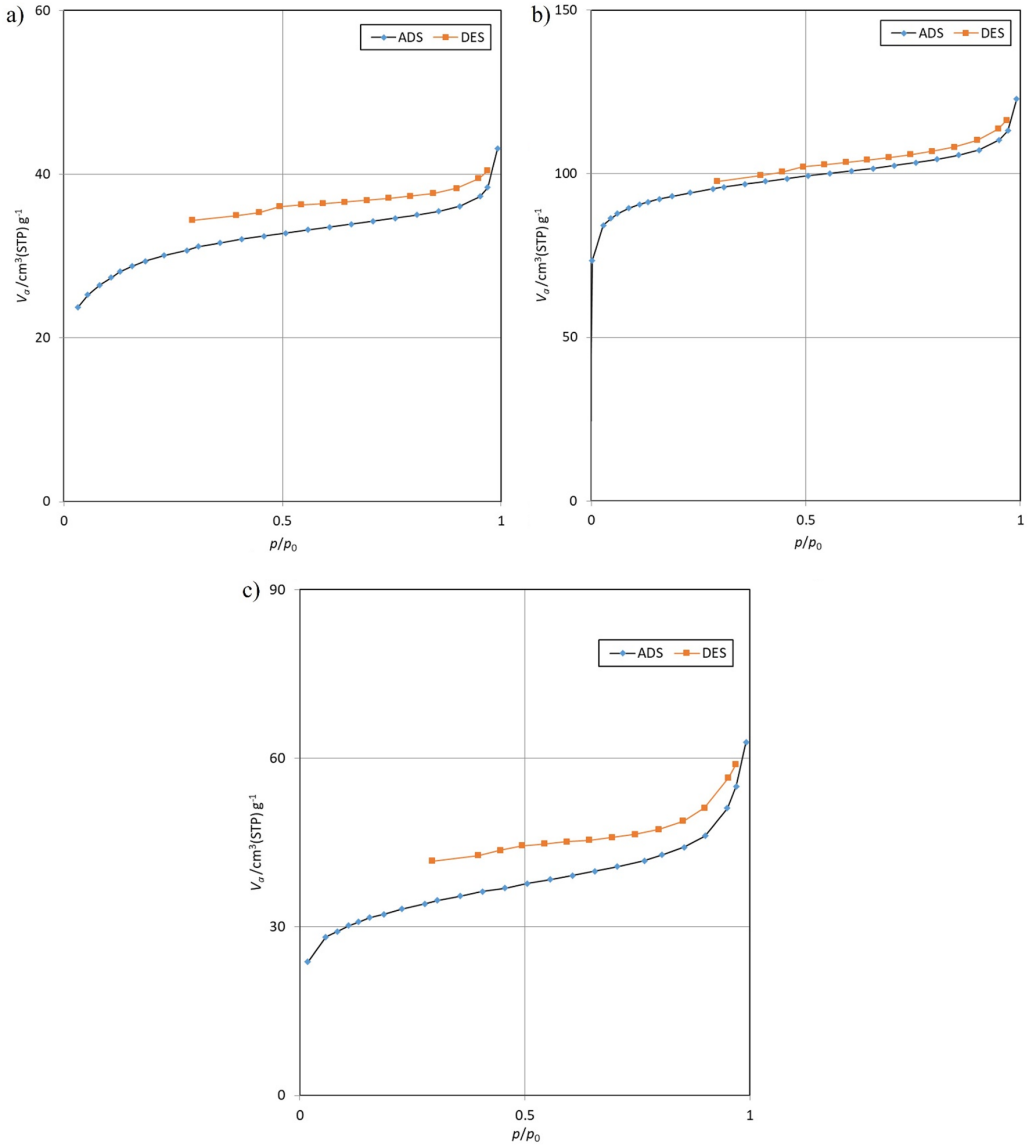
Adsorption and desorption diagrams for activated carbon obtained from (**a**) Bitter orange, (**b**) avocado, and (**c**) walnut.

The FESEM analysis involved capturing scanning electron photographs of the absorbent surface at varying distances, revealing changes brought about by the pyrolysis process. Images of all three adsorbers after undergoing pyrolysis are presented. Figure 2, displays images taken at a distance of 10 µm. Additional images captured at varying distances are included in the supplementary file (Figs. 1S–3S).

To examine the adsorbent material, we utilized the surface adsorption of nitrogen gas at a temperature of 77 K. The Brunauer–Emmett–Teller equation (BET) was utilized to compute the specific surface area of the adsorbent and the Barrett-Joyner-Hallenda (BJH) method to determine the total pore volume, particle size distribution, and surface area of fine pores. Table 3 summarizes the findings of the BET analysis^60, 61^.

**Table 3:** Surface characteristics and porosity of the three absorbents studied.

Adsorbent	Volume (cm^3^ (STP)/g)	BET (m^2^/g)	Total pore volume (cm^3^/g)	Average pore diameter (nm)
Avocado	82.839	360.55	0.1889	2.0952
Bitter orange	24.545	106.83	0.0669	2.4715
Walnut	26.891	117.04	0.0964	3.2967

Mesopores typically have diameters between 2 and 50 nm, making them larger than micropores but smaller than macropores. This classification is important because it influences the adsorption capacity and selectivity of the particles. By having a mesoporous structure, the adsorbent particles can provide a high surface area for adsorption, allowing them to effectively trap and retain molecules or substances within their pores. The data presented in Table 3. indicates that all three adsorbents are classified as mesoporous, in agreement with the previous studies^62, 63^. Figure 3, illustrates the nitrogen adsorption and desorption curves for the three adsorbents.

The data presented in Table 3 indicates that the biochar derived from avocado tree leaves has a significantly higher specific surface area in comparison to those obtained from Bitter oranges and walnuts.

**Figure 4:**
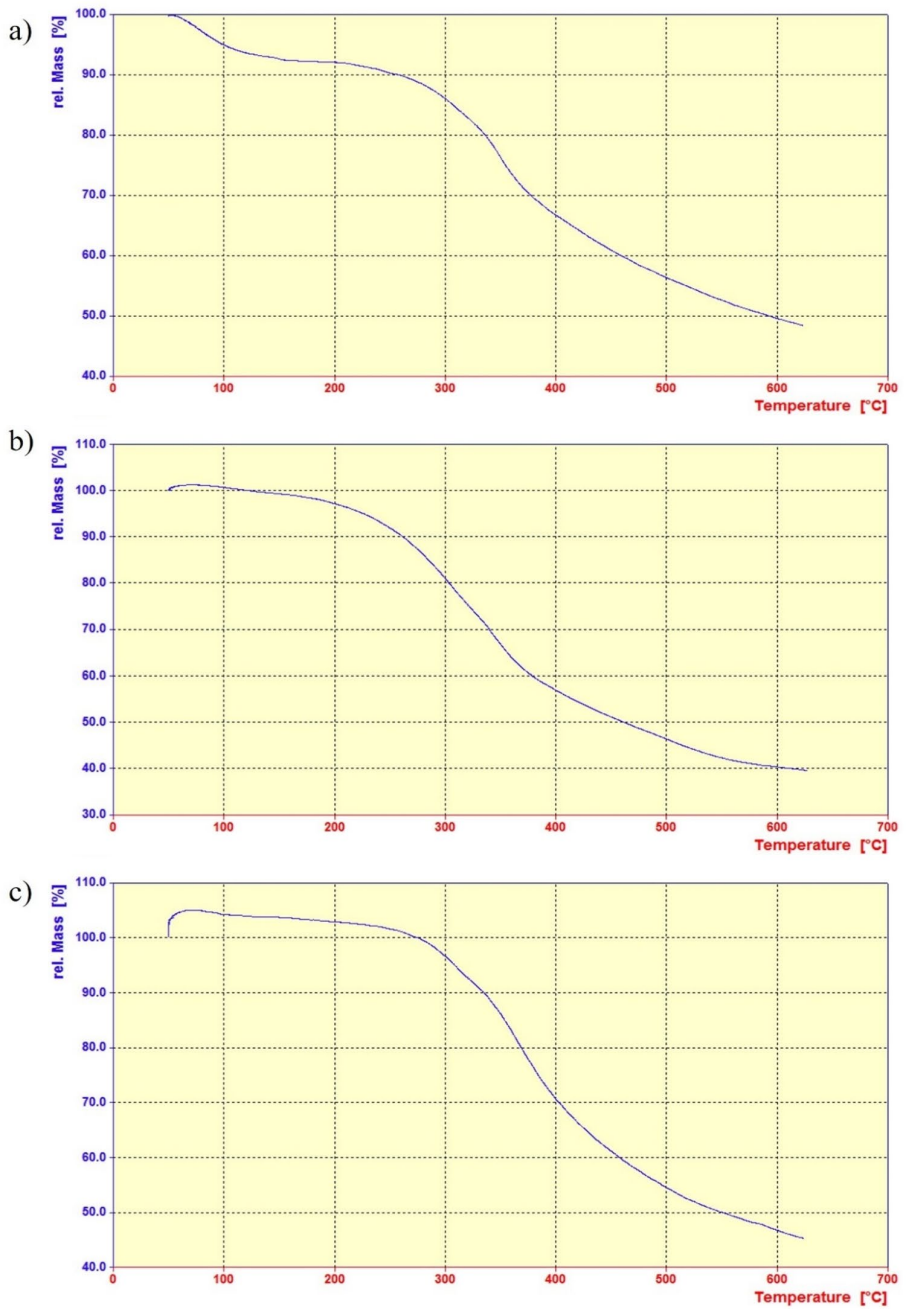
Thermogravimetric Analysis (TGA) graphs showcasing the pyrolytic behavior of leaves from: (**a**) Avocado, (**b**) Bitter orange, and (**c**) Walnut trees.

Thermogravimetric Analysis (TGA) is a type of analytical method that evaluates the thermal stability of a substance and determines its volatile content by measuring the sample's weight change when it is heated at a constant rate. To minimize energy and electricity consumption in the system, the pyrolysis temperature for all three biomasses was selected carefully. Therefore, the optimal temperature can only be determined via thermogravimetric testing and derivative thermogravimetric analysis (DTGA). The thermometric analysis of three types of biomass, namely avocado, Bitter orange, and walnut leaves, is presented in Fig. 4^64–66^.

### Effect of contact time

**Figure 5:**
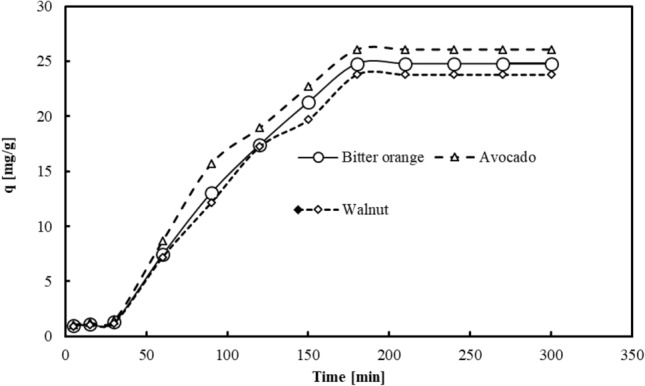
The impact of contact time on Pb^2+^ adsorption capacity of activated carbon derived from avocado, Bitter orange, and walnut leaves (initial pH 6.5, Pb concentration 50 ppm, adsorbent dose 1.5 g/L and temperature 25 °C).

Figure 5, displays the results of the investigation into the impact of contact time on Pb^2+^ removal using activated carbon derived from the pyrolysis of avocado, walnut, and Bitter orange leaves. The outcomes suggest that the adsorption capacity for Pb^2+^ rises as the contact time increases. However, after a certain duration, this trend slows down and eventually reaches equilibrium, with no notable change in the adsorption rate. Therefore, the optimal time required to achieve equilibrium conditions for the Pb^2+^ adsorbate is 3 h for all three adsorbents.

The initial rapid adsorption can be attributed to the strong affinity and reactivity between the adsorbent and Pb^2+^. The rapid adsorption may be attributed to the existence of numerous adsorption sites and a mass transfer concentration gradient between the adsorbent and adsorbate^67, 68^. Towards the conclusion of the reaction, the adsorption rate declined since the adsorption sites became saturated, and the number of available spaces for adsorption decreased^69^. Activated carbon generated from avocado, Bitter orange, and walnut leaves demonstrated maximum adsorption capacity at equilibrium of 26.07, 24.77, and 23.78 mg/g, respectively, at 50 ppm. The variation in the Pb^2+^ adsorption rate may be attributed to differences in the molecular structure, spatial effects, and porosity of the adsorption medium^68^.

### Effect of solution pH

**Figure 6:**
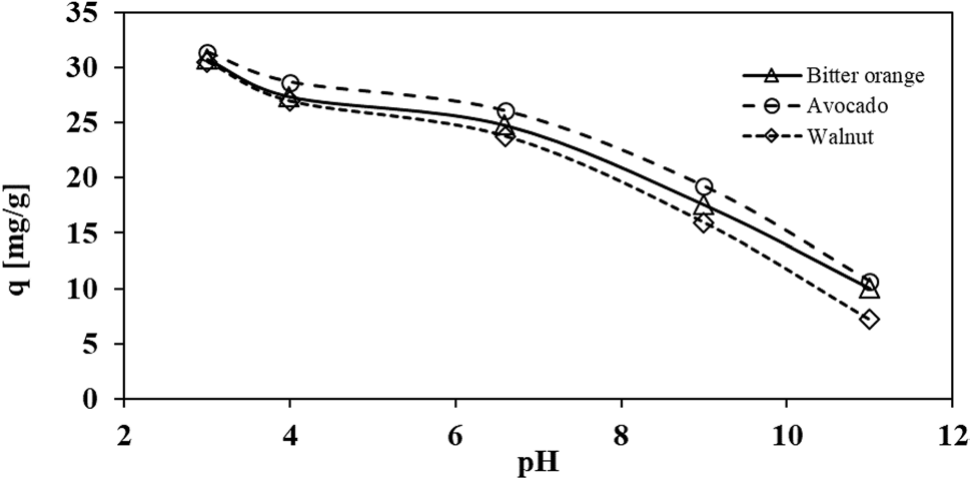
The impact of pH at 25 °C and an optimal time of 180 min on the Pb adsorption capacity of activated carbon derived from avocado, Bitter orange, and walnut leaves.

Figure 6, depicts the influence of pH on the adsorption capacity of Pb^2+^ using activated carbon derived from avocado, Bitter orange, and walnut leaves. The results demonstrate that the adsorption mechanisms of all three adsorbents exhibit relatively similar behavior when in contact with Pb^2+^. At lower pH levels, the protonation of the solution causes Pb^2+^ to be more readily degraded or attached to the adsorbent surface due to the existence of H^+^ in the solution, in addition to adsorption by the activated carbon, leading to greater Pb^2+^ degradation or removal. As the acidity of the solution decreases or the pH increases, the competition between the functional groups in Pb^2+^ and OH^-^ ions for adsorption on the adsorbent increases, and the metal cannot be absorbed as easily as before.

The maximum capacity for metal adsorption was observed at a pH of 3. When the pH was increased from 3 to 11, the activated carbon derived from avocado, Bitter orange, and walnut leaves showed an increase in adsorption capacity for metals, ranging from 31.39 to 10.64 mg/g for avocado, 30.78 to 98.00 mg/g for Bitter orange, and a decrease from 30.52 to 7.20 mg/g for walnut. These results indicate that the three absorbers exhibited similar behavior when in contact with Pb^2+^.

### Effect of adsorbent dosage

**Figure 7:**
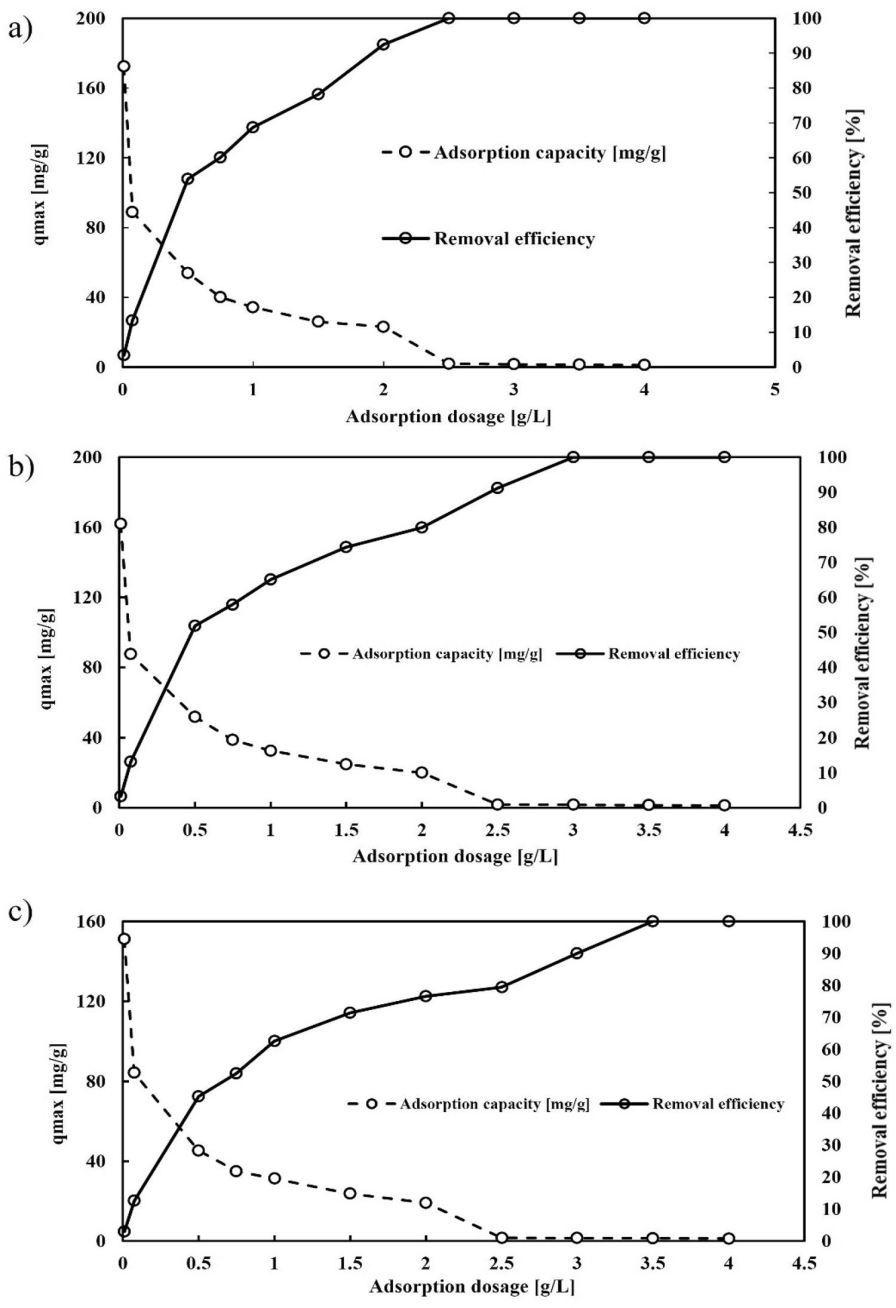
Effect of adsorption capacity and Pb^2+^ removal efficiency of (**a**) avocado leaf adsorbent, (**b**) bitter orange leaf adsorbent, and (**c**) walnut leaf adsorbent (contact time of 12 h, initial pH of 6.5, Pb concentration of 50 ppm, and temperature of 25 °C).

Figure 7, depicts the impact of the adsorbent dosage on both the adsorption capacity and removal efficiency of Pb^2+^. The data and diagrams indicate that increasing the amount of adsorbent used in the experiments significantly increases the removal efficiency. For instance, Fig. 7, shows that the activated carbon derived from pyrolysis of avocado leaves can remove Pb^2+^ from 3 to 100% by incrementing the adsorbent dosage from 0.01 to 4 g/L in the same volume of solution. The comparison of different adsorbents revealed that the avocado leaf adsorbent offered high removal efficiency with less material, likely attributed to its significant porosity, small cavities, and functional groups on the surface.

The adsorption capacity decreases as the adsorbent dosage increases in all cases due to two factors. Initially, an increase in adsorbent dosage at a constant concentration of the metal solution Pb2 + s to an increase in unsaturated adsorption sites. However, an increase in adsorbent dosage results in a decrease in equilibrium adsorption capacity and the overlap of adsorption sites due to the adsorbent's concentration^70, 71^.

### Effect of concentration

**Figure 8:**
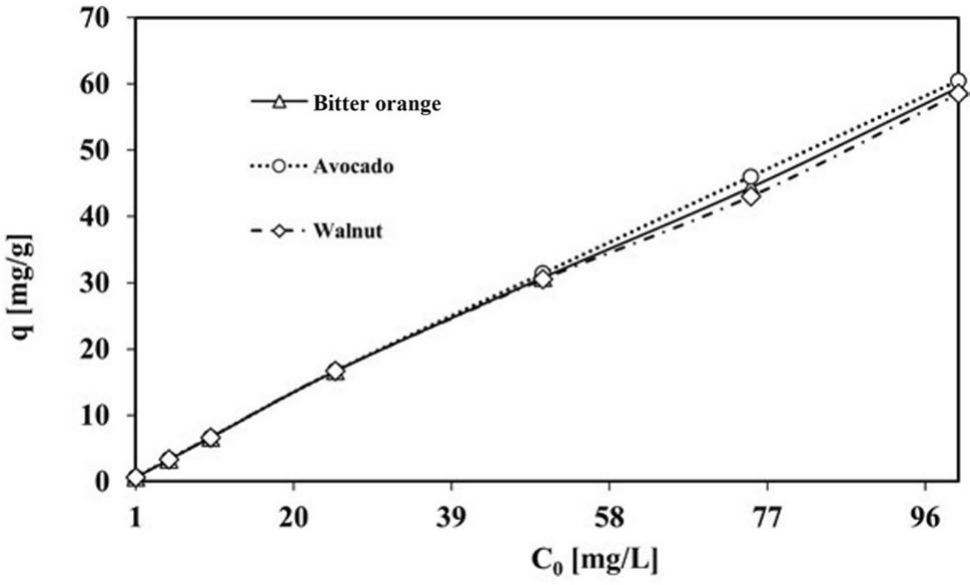
The impact of the initial solution concentration on the Pb^2+^ adsorption capacity of activated carbon produced from avocado, Bitter orange, and walnut leaves (contact time: 24 h, pH = 3, adsorbent dosage: 1.5 g/L, temperature: 25 °C).

Figure 8, shows that the Pb^2+^ level refers to the amount of Pb^2+^ that is adsorbed by the adsorbent per unit of its mass increases as the driving force for mass transfer between the solution and adsorbent phases increases, enabling the overcoming of mass transfer resistance.

Figure 8, demonstrates the effect of the Pb solution concentration on the adsorption capacity of the three adsorbents at various concentrations. Figure 8, shows that the Pb^2+^ level this refers to the amount of Pb^2+^ that is adsorbed by the adsorbent per unit of its mass increases as the driving force for mass transfer between the solution and adsorbent phases increases, enabling the overcoming of mass transfer resistance. There is also a greater likelihood of collisions between metal and adsorbent molecules with an increasing initial concentration^72^.

### Effect of temperature

**Figure 9:**
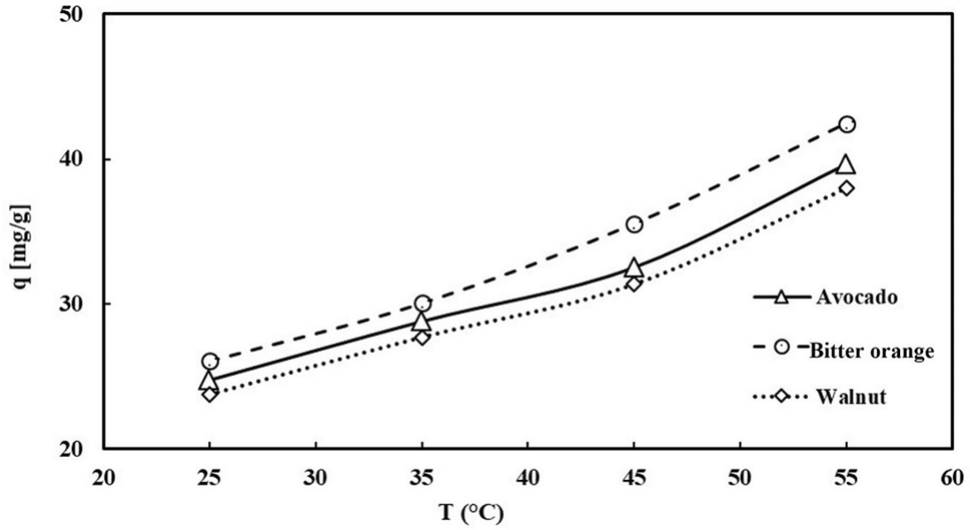
Temperature effects on Pb^2+^ adsorption by avocado, Bitter orange, and walnut activated carbon (adsorbent contact time: 3 h, initial pH = 6.5, Pb concentration 50 ppm).

Figure 9, depicts the impact of temperature on Pb^2+^ adsorption levels. The findings reveal that with the rise in temperature, the capacity for adsorption also increases. This phenomenon can be ascribed to three factors: the segregation of functional groups on the surface, an augmentation in surface-active sites, and a reduction in the thickness of the boundary layer encompassing the adsorbent. The experiment was carried out at four distinct temperatures, namely 25 °C, 35 °C, 45 °C, and 55 °C. The capacity of three adsorbents to adsorption derived from avocado, Bitter orange, and walnut leaves increased from 26.07 to 42.45 mg/L, 24.77 to 39.65 mg/L, and from 23.78 to 37.98 mg/L, respectively.

### Theory of adsorption, kinetics, isotherms

To investigate the factors that Influence the rate at which adsorption occurs, we conducted a study on adsorption kinetics. We utilized the pseudo-first-order and pseudo-second-order kinetic models, along with intra-particle diffusion models, to determine the appropriate kinetic model. The results, including the parameters that were computed for any model, are reported in Table 4. By evaluating the correlation coefficients (R^2^) of the kinetic equations, we determined that the pseudo-second-order equation provided an accurate fit for the in vitro data of all three adsorbents in each case, based on the obtained data. Furthermore, we found that the calculated equilibrium capacity (q_e,calc_) derived from the pseudo-first-order equation matched better with the equilibrium capacity (q_e,exp_) measured in experiments. This suggests that the rate of adsorption is primarily dependent on the quantity of vacant sites on the adsorbent. Additionally, it appears that the decisive step in the adsorption process may involve the exchange or sharing of electrons between the adsorbent and the adsorbate.

**Table 4:** Constants for equations of kinetic in all experiments.

Adsorbent	Active carbon derived from avocado leaf	Active carbon derived from Bitter orange leaf	Active carbon derived from walnut leaf
Adsorbate	Pb	Pb	Pb
q_e,exp_ (mg/g)	31.39	30.78	30.52
Pseudo-first-order kinetic			
q_e,calc_ (mg/g)	32.38	31.30	29.23
k_1_ (L/min)	− 0.0136	0.0129	0.0122
R^2^	0.9543	0.9367	0.9543
Pseudo-second-order kinetic			
q_e,calc_ (mg/g)	30.58	29.67	28.49
k_2_ (g/mg min)	0.0007	0.0006	0.0006
R^2^	0.971	0.9623	0.9663
Intra-particle diffusion kinetic			
K_id_ (mg/g min^1/2^)	61.06	56.43	53.15
* I*	0.1513	0.1666	0.1759
R^2^	0.7893	0.7709	0.7726

Adsorption capacity of adsorbents can be approximated by analyzing the equilibrium sorption isotherms. This capacity value can serve as a basis for designing a commercial treatment system. Adsorption isotherms delineate the equilibrium connection between the adsorbate and adsorbent, which can be characterized by the Langmuir, Freundlich, and Temkin isotherms^73, 74^.

At present, linearization followed by linear regression is a widely adopted method for predicting isotherm parameters. However, linearization of equations can Pb^2+^ to a decrease in the correlation coefficient (R^2^) during linear studies. To mitigate this issue, non-linear regression is utilized to obtain isotherm parameters without the natural bias induced by linearization^75^. The most efficient method of calculating isotherm equations is the non-linear analytic mathematical approach, which is also capable of predicting sorption performance under varying operating conditions. The method of least squares is commonly used by researchers to determine the best fit for adsorption isotherms. However, for selecting the optimal isotherm, non-linear regression is deemed to be the most effective method. This approach minimizes errors in the relationship between equilibrium data and the values predicted by isotherms^76–78^.

In this research, we analyzed Langmuir, Freundlich, and Temkin isotherms using in vitro data under optimal conditions. As can be observed from Table 5, the experimental data fits better with the Freundlich isotherm for Pb^2+^ removal with the activated carbon from avocado, Bitter orange, and walnut leaves, whereas the Langmuir and Temkin isotherms show relatively less accurate fits. To provide a more comprehensive explanation and understanding of this issue, we have included the results of Langmuir, Freundlich, and Temkin isotherms, along with the nonlinear error analysis of each isotherm in Table 5. Supplementary file contains non-linear graphs pertaining to adsorption isotherms for all three adsorbents (Figs. 4S–12S).

**Table 5:** Nonlinear isotherms constants and the calculated error rate of isothermal models.

Adsorbent	Active carbon derived from avocado leaf	Active carbon derived from Bitter orange leaf	Active carbon derived from walnut leaf
Langmuir isotherm			
q_m_ (mg/g)	106.57	119.25	95.86
k_1_ (L/min)	0.13598	0.08272	0.10202
R^2^	0.9969	0.98139	0.96025
Freundlich isotherm			
K_F_ (mg/g) (L/mg)^1/n^	16.83	12.39	12.68
1/n	0.570	0.638	0.5757
R^2^	0.99945	0.98651	0.96615
Temkin isotherm			
b_T_(J/mol)	24.47	24.95	21.829
K_T_(L/g)	1.19808	0.856	0.92031
R^2^	0.99581	0.9786	0.95962

## Conclusion

This research aimed to investigate the development and synthesis of water-repellent adsorbents for the effective adsorption of metal-containing solutions, specifically targeting Pb^2+^. The choice of this particular metal was based on its significant global consumption and its presence in metal and industrial waste. By utilizing the cost-effective pyrolysis process and activating the biomass with sulfuric acid, the temperature rate reduction during pyrolysis resulted in increased porosity, thereby enhancing the adsorption potential. Thermogravimetric analysis of avocado, bitter orange, and walnut leaf biomasses revealed that temperatures exceeding 600 °C led to higher energy consumption without significant mass reduction, indicating no further reactions or improvements. The key findings are as follows: Avocado leaves demonstrated higher adsorption capacity for Pb^2+^. The adsorption of Pb^2+^ by activated carbon derived from avocado, bitter orange, and walnut leaves increased with longer contact time, lower solution pH, higher Pb^2+^ concentration, and higher temperature. However, increasing the quantity of adsorbent utilized resulted in decreased adsorption capacity. According to the Freundlich model, the maximum adsorption capacity of Pb^2+^ by activated carbon derived from avocado leaves was found to be 96.15 mg/g. Additionally, the adsorption of Pb^2+^ on avocado leaf-derived activated carbon fitted well with the pseudo-second-order kinetics. Despite having a smaller average pore diameter compared to orange and walnut leaves, the activated carbon derived from avocado leaves exhibited greater adsorption of Pb^2+^ than the other two types. Furthermore, a detailed comparison of the lignocellulosic structures, adsorption capacity, and quantity among the three similar biomasses indicated that the adsorbent with a higher hemicellulose content (avocado leaf) showed a higher percentage of adsorption. This is attributed to the functional groups on the surface, the type of metal, and the higher specific surface area. In conclusion, this study shed light on the potential of hydrophobic adsorbents for the efficient removal of metal ions from solutions. Avocado leaf-derived activated carbon showed superior performance in adsorbing Pb^2+^. The findings provided valuable insights into the adsorption mechanisms, optimizing process conditions, and the importance of biomaterial composition in designing effective adsorbents for environmental remediation.

### Supplementary Information

Supplementary Figures.

## Data Availability

All data generated or analyzed data for the experimental part of this study are included in this published article. The data that support the findings of this study are available from the corresponding author, [Salman Movahedirad], upon reasonable request. Moreover, all other data that support the plots within this paper and other finding of this study are available from the corresponding author upon reasonable request.
