# Supplementary material for: Synthesis and characterization of cost-effective and high-efficiency biochar for the adsorption of Pb2+ from wastewater
Source: Sci Rep. 2023 Sep 20;13:15608. doi: 10.1038/s41598-023-42918-0 (PMC10511742; doi:10.1038/s41598-023-42918-0)
Supplement: Supplementary file 1 — Supplementary Figures. [file 41598_2023_42918_MOESM1_ESM.docx]

**Supplementary Information**

**Synthesis and characterization of cost-effective and high-efficiency biochar for the adsorption of lead ions from wastewater**

Hatef Bassareh, Masoud Karamzadeh, Salman Movahedirad*

Department of Chemical Engineering, Iran University of Science & Technology, Tehran, Iran.

Corresponding author: Salman Movahedirad (*E-mail address*: [movahedirad@iust.ac.ir](mailto:movahedirad@iust.ac.ir))


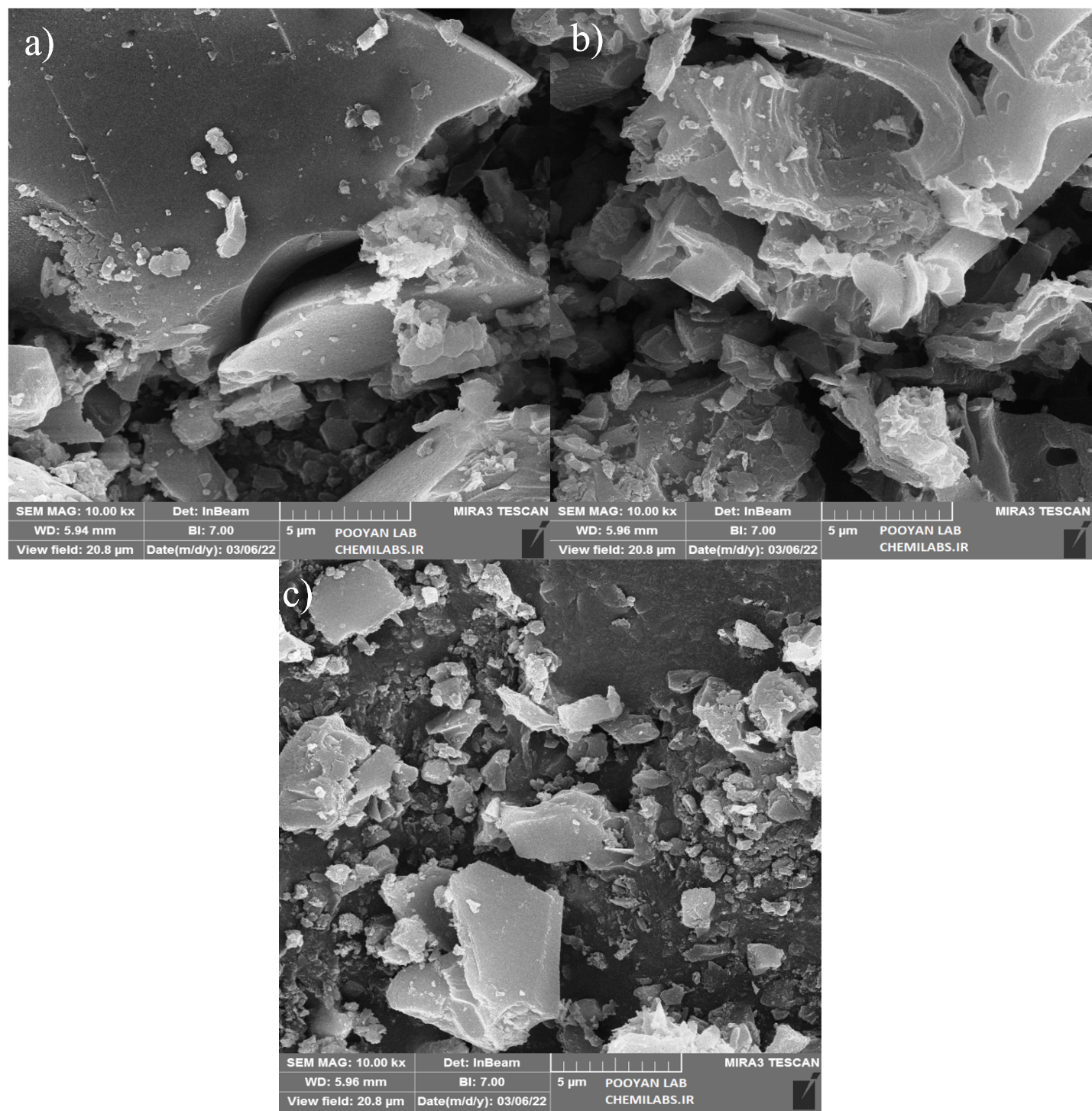


**Figure 1S.** Image of a) Bitter orange, b) avocado, and c) walnut biochar from a distance of 5 micrometers.


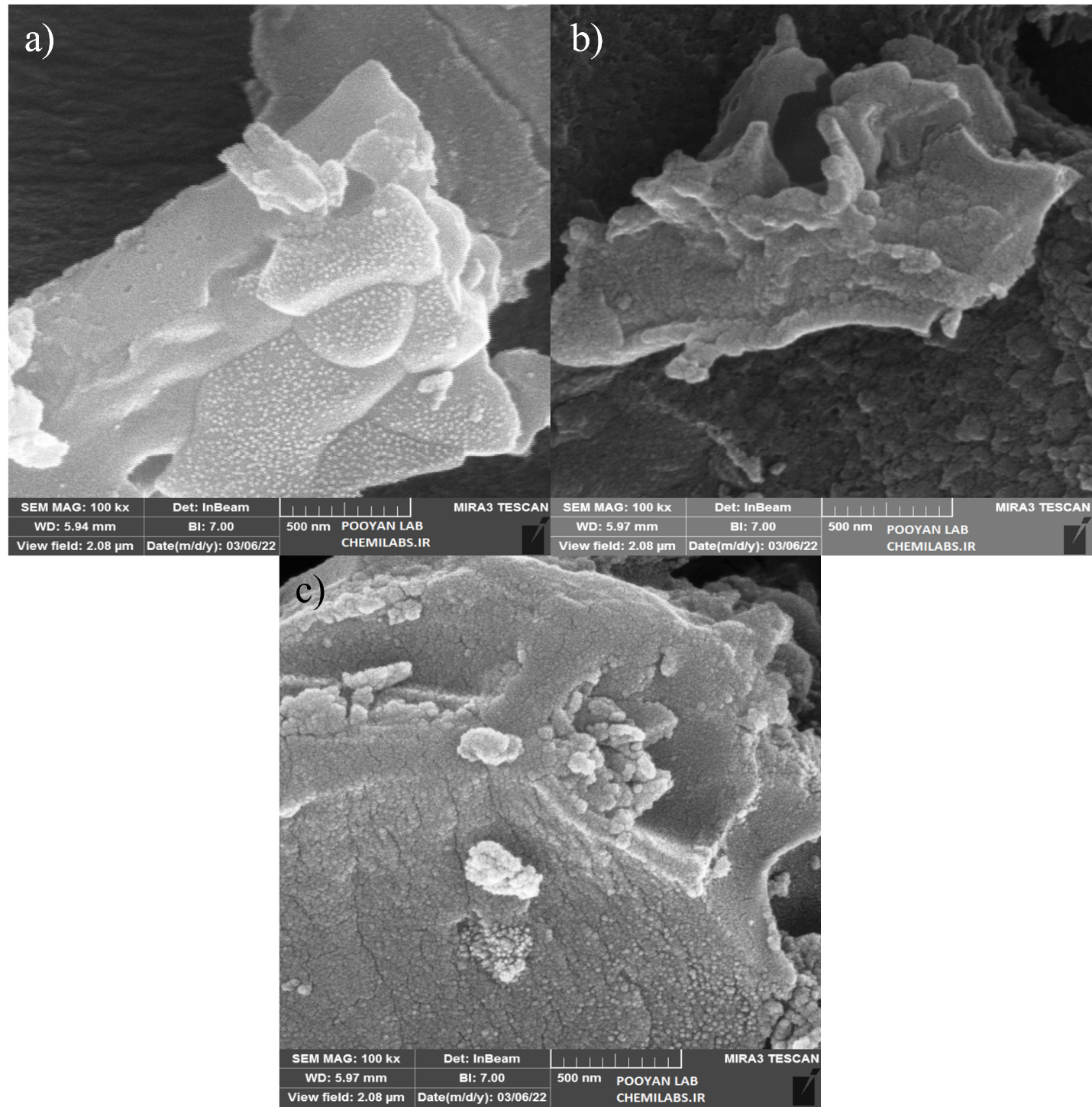


**Figure 2S.** Image of a) Bitter orange, b) avocado, and c) walnut biochar from a distance of 500 nanometers.


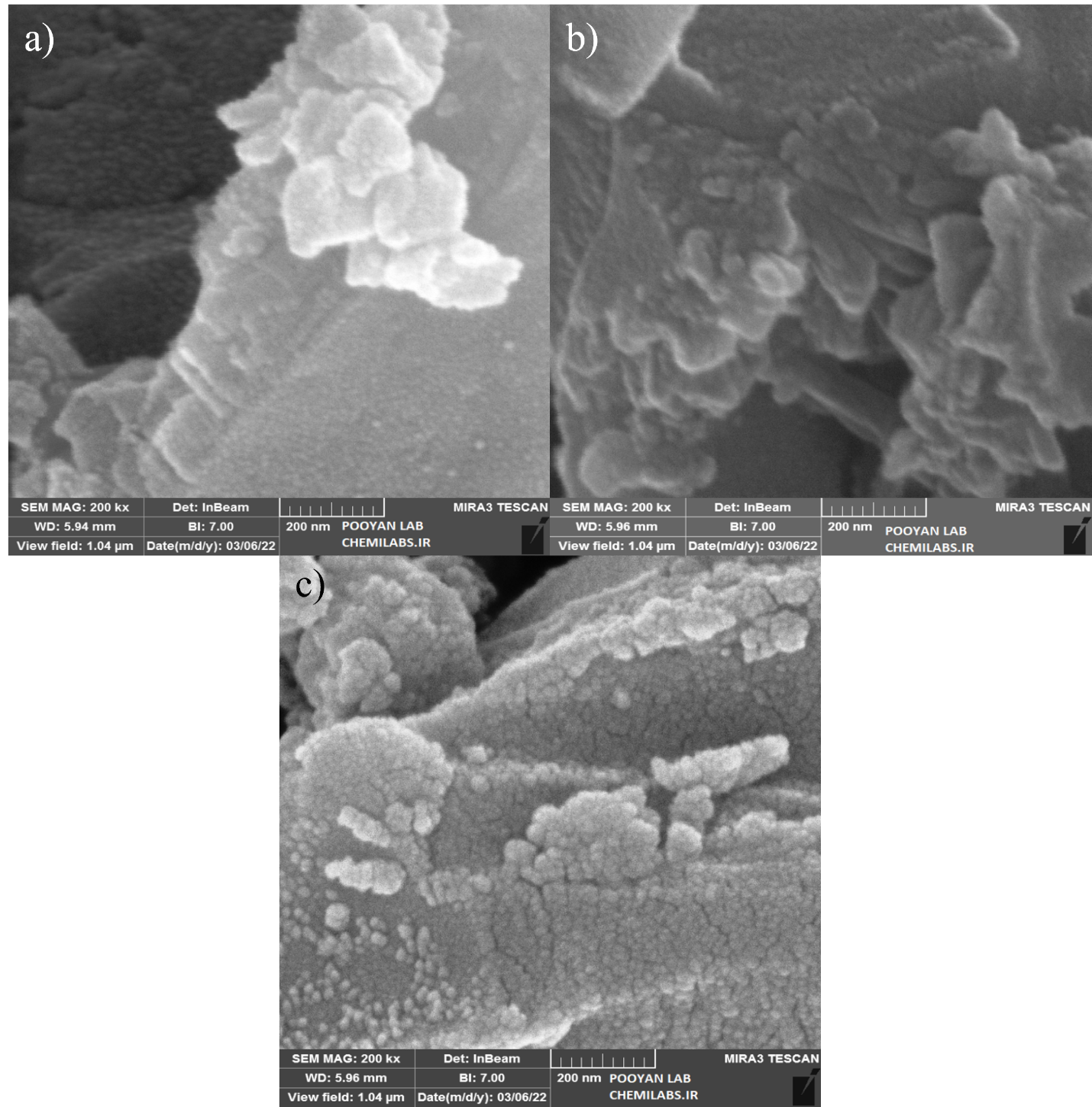


**Figure 3S.** Image of a) Bitter orange, b) avocado, and c) walnut biochar from a distance of 200 nanometers.


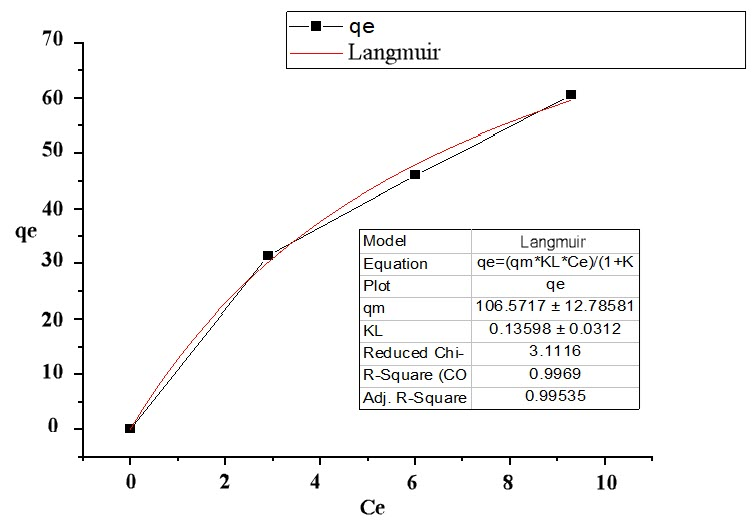


**Figure 4S.** Nonlinear plots of Langmuir isotherms for avocado leaves.


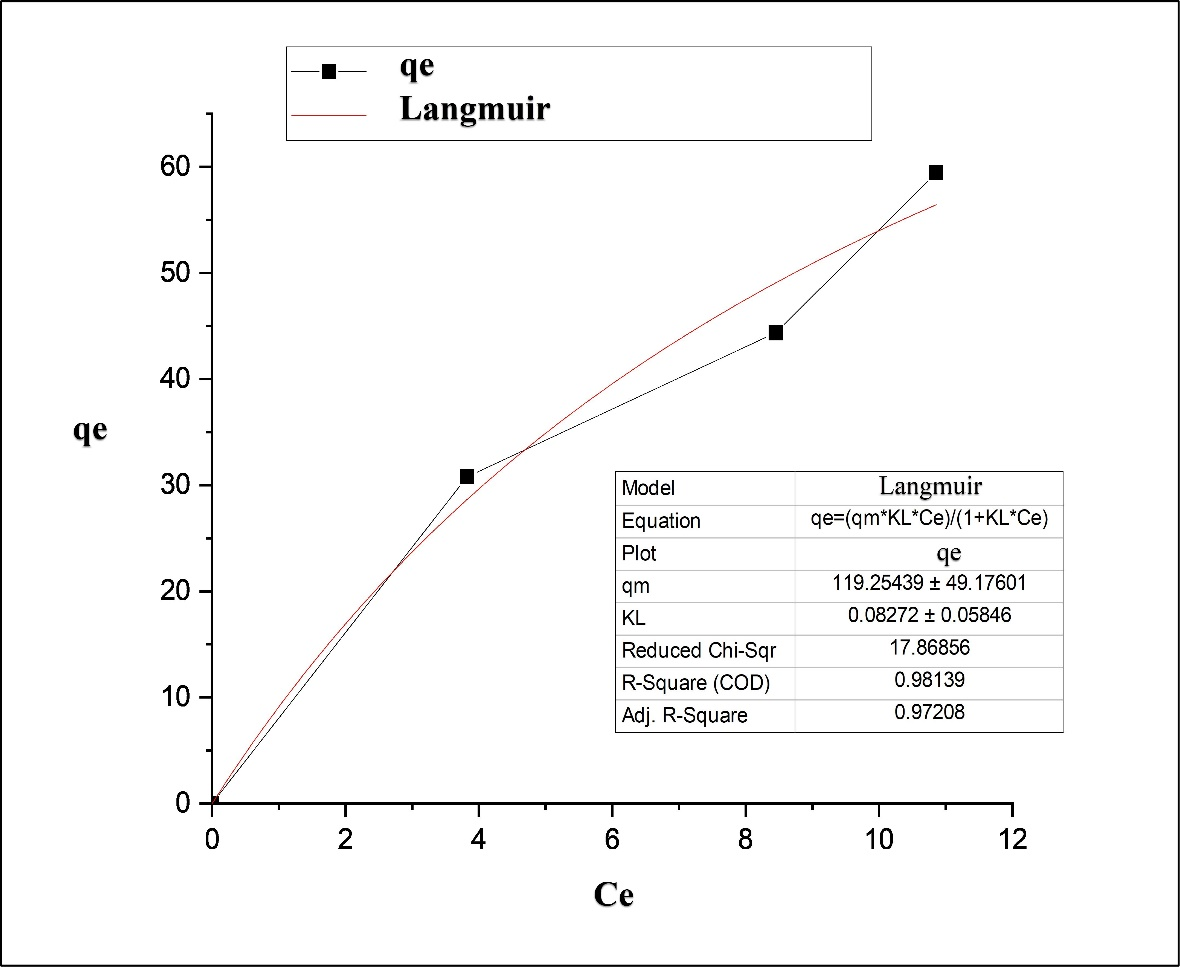


**Figure 5S.** Nonlinear plots of Langmuir isotherms for bitter orange leaves.


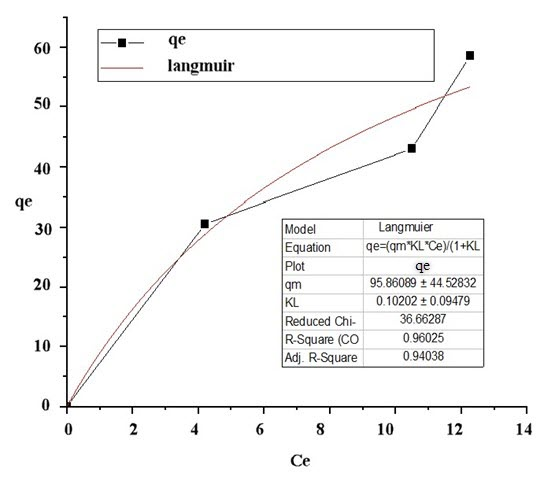


**Figure 6S.** Nonlinear plots of Langmuir isotherms for walnut leaves.


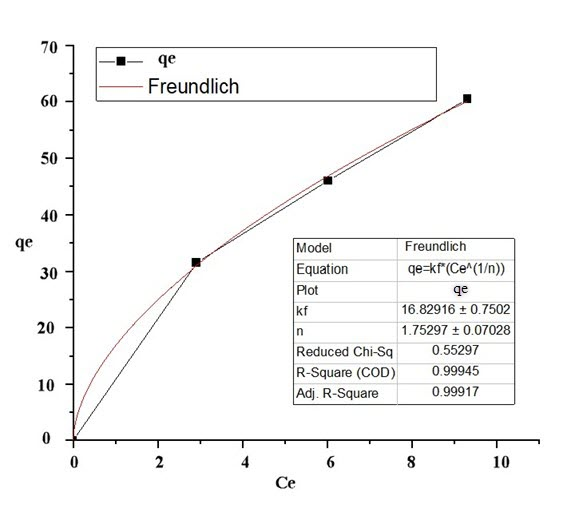


**Figure 7S.** Nonlinear plots of Langmuir isotherms for avocado leaves.


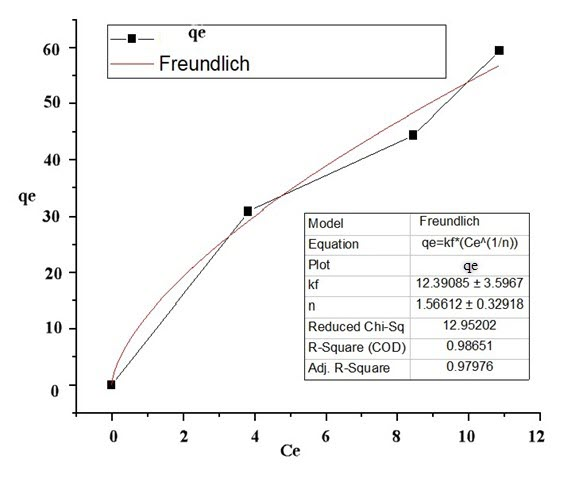


**Figure 8S.** Nonlinear plots of Freundlich isotherms for bitter orange leaves.


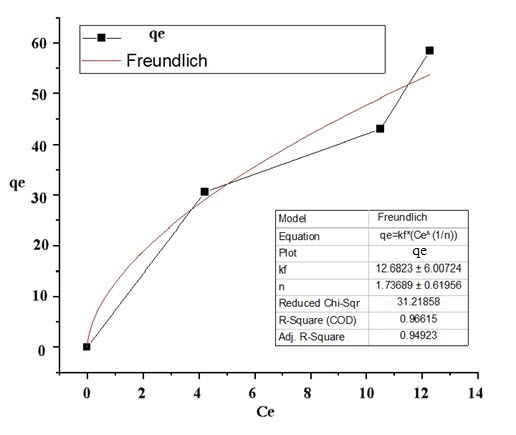


**Figure 9S.** Nonlinear plots of Freundlich isotherms for walnut leaves.


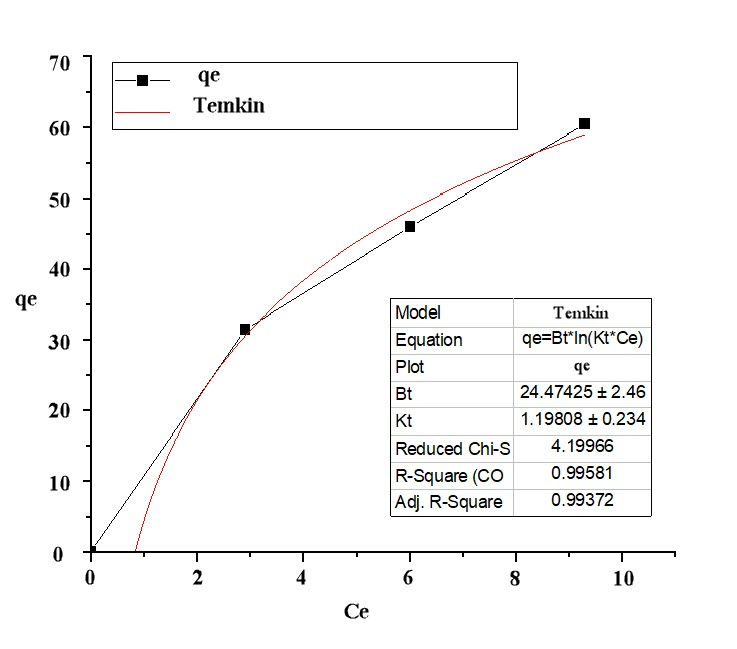


**Figure 10S.** Nonlinear plots of Temkin isotherms for avocado leaves.


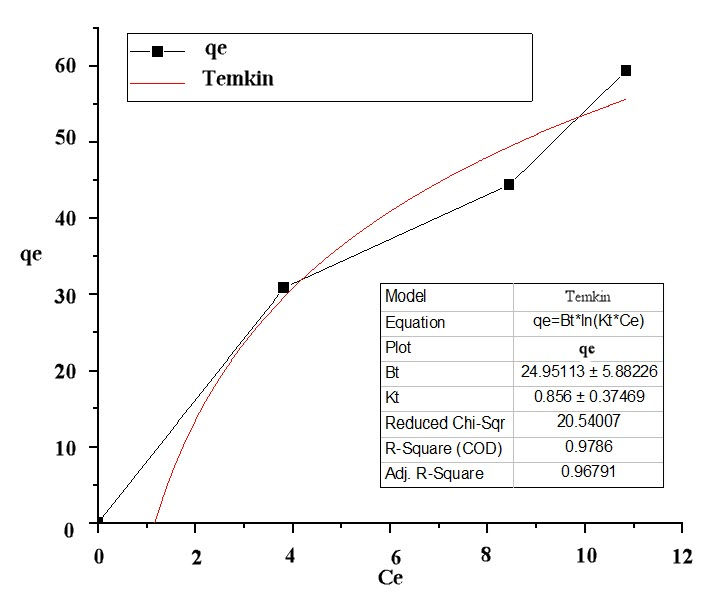


**Figure 11S.** Nonlinear plots of Temkin isotherms for bitter orange leaves.


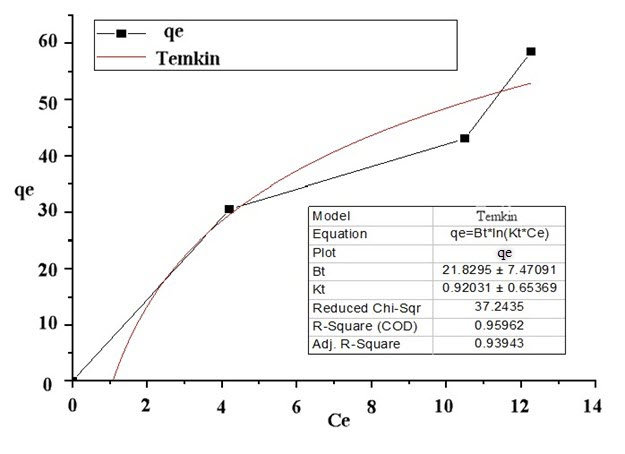


**Figure 12S.** Nonlinear plots of Temkin isotherms for walnut leaves.
